# Supplementary material for: Comprehensive tool for a phase compensation reconstruction method in digital holographic microscopy operating in non-telecentric regime
Source: PLoS One. 2023 Sep 8;18(9):e0291103. doi: 10.1371/journal.pone.0291103 (PMC10491004; doi:10.1371/journal.pone.0291103)
Supplement: S1 File — (DOCX) [file pone.0291103.s001.docx]

**User Manual for The NoTele DHM (noteleDHM) reconstruction algorithm**

Non-Telecentric DHM reconstruction algorithm (noteleDHM) – Version 1.0

Brian Bogue-Jimenez, Carlos Trujillo and Ana Doblas

March 2023

Contact: [catrujilla@eafit.edu.co](mailto:catrujilla@eafit.edu.co), [adoblas@memphis.edu](mailto:adoblas@memphis.edu), opticalimagingresearchlab@gmail.com

OIRL, Department of Electrical and Computer Engineering, University of Memphis, Memphis T.N.

Applied Optics Research Group, School of Applied Sciences and Eng., Universidad EAFIT, Medellin, Colombia.​

​

Content

| 1. About noteleDHM………………………………………………………………………………………… | 4 |
| --- | --- |
| 1.1 Credits………………………………………………………………………………………………………… | 4 |
| 1.2 Citation……..…………..…………………………………………………………………………………… | 4 |
| 1.3 Support……..…………..…………………………………………………………………………………… | 4 |
|  |  |
| 1. Running noteleDHM……………………………………………………………….…………………….. | 4 |
| 2.1 Raw version………………………………………………………………………………………………… | 4 |
| 2.1.1 read the hologram………………………………………………………………………………. | 5 |
| 2.1.2 input parameters………………………………………………………………………………… | 5 |
| 2.2 GUI version………………………………………….……………………………………………………… | 6 |
| 2.2.1 installation…………….……………………………………………………………………………. | 6 |
| 2.2.2 running GUI version ………………..………………….………………………………………. | 6 |
| 1. Usage example……………………………………………………………………………………………… | 7 |
| 1. References……………………………………………………………………………………………………. | 8 |

1. **About noteleDHM**

noteleDHM is a MATLAB and Python implementation developed collaborating between the Optical Imaging Research Laboratory (Department of Electrical and Computer Engineering at the University of Memphis) and the Applied Optics Research Group (School of Applied Sciences and Eng., Universidad EAFIT, Medellin, Colombia). noteleDHM MATLAB App is a free, user-friendly tool, providing a streamlined strategy for reconstructing off-axis holograms recorded using a digital holographic microscope (DHM) operating in non-telecentric mode. This implementation is based on a spectral method [1] in combination with a minimization algorithm that finds the parameters of the spherical wavefront to reconstruct a quantitative phase image without or minimum phase perturbations due to the interference angle of the off-axis configuration and the non-telecentric configuration in the DHM imaging system. The input parameters of the noteleDHM method are the sensor's pixel size and the wavelength of the light source. The noteleDHM computational algorithm offers an open-source reconstruction tool for the DHM community.

- 1. **Credits**
- noteleDHM is developed in MATLAB 2021a (version 9.19.0, R2021a, Natick, Massachusetts: The MathWorks Inc.) and Python 3.7.1 (2018).
- For the unwrapping step, nonteleDHM implements the code developed by Herráez *et.al*. [2,3]
  1. **Citation**

If using noteleDHM for publication, please kindly cite the following:

- B. Bogue-Jimenez, C. Trujillo, and A. Doblas, “Comprehensive Tool for a Phase Compensation Reconstruction Method in Digital Holographic Microscopy Operating in Non-Telecentric Regime,” Computer Physics Communications, *under review* (2023).

**1.3 Support**

If you use noteleDHM and find a bug, please contact us via email and we will address the problem. Our emails are [opticalimagingresearchlab@gmail.com](mailto:opticalimagingresearchlab@gmail.com), [catrujilla@eafit.edu.co](mailto:catrujilla@eafit.edu.co), [adoblas@memphis.edu](mailto:adoblas@memphis.edu).

1. **Running noteleDHM**

There are presently two versions of noteleDHM. The initial one comprises of the raw MATLAB and Python scripts, while the second one is a user-friendly MATLAB GUI version.

- 1. **Raw version**

The target audience for this version is coding developers or imaging researchers who possess proficiency in both Python and MATLAB. The scripts are available for download at <https://github.com/OIRL/noteleDHM-Tool>. Upon downloading and unzipping the repository files, the user will have access to a folder that includes four MATLAB and Python scripts (no_tele_main.py, no_tele_main.m, funs.py, and funs.m) and two subfolders (data and MATLAB GUI). **Section 2.2** delves into the details of the MATLAB GUI. The following subsections provide an overview of the fundamental use and functionality of the two raw implementations.

- - 1. **MATLAB scripts**

To execute the phase compensation method, use the 'no_tele_main.m' script, which is the primary operating script, while the 'funs.m' script contains the necessary auxiliary functions. When running the 'no_tele_main.m' script, pay attention to the following lines:

% Loading image file (hologram) to process

user_input = '-4cm_20x_star.tiff';

filename = ['data/', user_input];

disp(['Non-telecentric DHM hologram: ', filename]);

% Variables and flags for hologram reconstruction (Default variables, change accordingly)

Lambda = 633*10^(-9);

dx = 6.9*10^(-6);

dy = 6.9*10^(-6);

%Different available optimization methods (only needed for automatic method)

%1: FMC 2: FMU 3: FSO 4: SA 5: PTS 6: GA 7: PS 8: GA+PS (See documentation for further details)

algo = 8; %Select method as desired

%Two available cost functions (only needed for automatic method)

%cost = 1 # 0 - BIN -- 1 - SD (See documentation for further details)

cost = 1; %Select function as desired

When using the 'no_tele_main.m' script, ensure that you enter the name of the hologram file you wish to process as 'user_input'. The file must be located inside the 'data' folder, which should be in the current directory.

'Lambda' is a variable used to input the illumination wavelength used for hologram recording, while 'dx' and 'dy' are input variables that represent the pixel pitch of the camera sensor.

'algo' is a variable flag that lets you select from different available optimization algorithms for phase compensation. This variable is only applicable when running the script in "automatic mode," as explained in section **2.1.1.1.** The available optimization methods are:

1. FMC method finds the minimum of a constrained multivariable function. fmincon in MATLAB and NonlinearConstraint in Python’s scipy.

2. FMU approach finds the minimum scalar value of a non-linear unconstrained multivariable objective function. fminunc in MATLAB, and fmin_ncg in Python’s scipy.

3. FSO solver returns a vector that minimizes the objective function by solving for the function F(x) = 0. fsolve in MATLAB and fsolve in Python’s scipy.

4. SA is a simulated annealing probabilistic technique well suited for finding the global minimum of a large and discrete search space. Simmulan-nealbnd in MATLAB and dual_annealing in Python’s scipy.

5. PTS algorithm that finds the points in a Pareto front that minimizes two cost functions of a two-dimensional variable. In this case, we use the J1 and J2 cost functions. paretosearch in MATLAB and basinhopping from Python’s scipy.

6. GA algorithm minimizes a cost function given the number of variables in the function by iteratively picking the best population values within the range specified by the bounds. ga in MATLAB and differential_evolution in Python’s scipy.

7. PS algorithm that does not utilize gradients, allowing for the convergence of cost functions that are not continuous or differentiable. patternsearch in MATLAB and minimize with Nelder-Mead method in Python’s scipy.

The 'cost' variable flag allows you to choose from various available cost functions for the optimization stage. This variable is only relevant when running the script in "automatic mode," which is described in section 2.1.1.1. The available cost functions are:

BIN is a metric that involves thresholding and summation. It takes into account the number of phase jumps within the phase image. The metric is defined as:


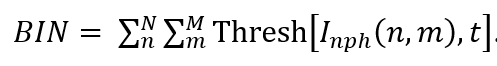


Here, *M* and *N* represent the total number of pixels in the image in each direction. 'I_nph_' denotes the phase image, while 'n' and 'm' are integers that indicate the pixel locations throughout the image. 't' is the thresholding factor used in the calculation.

Lastly, the user must choose which compensation mode to execute.

%{

0: Manual determination of the M&N and H&G coordinates for no-tele compensation.

1: Automatic determination of these parameters.

%}

auto = 0;

To choose between the manual or automatic determination of the +1 diffraction order Region of Interest and the center of the remaining spherical phase factor, set the 'auto' variable flag to either 0 or 1. A value of 1 indicates automatic determination, while a value of 0 indicates manual determination.

- - - 1. **Automatic mode**

In this mode, the user is presented with several intermediate images. As depicted in the following figure.


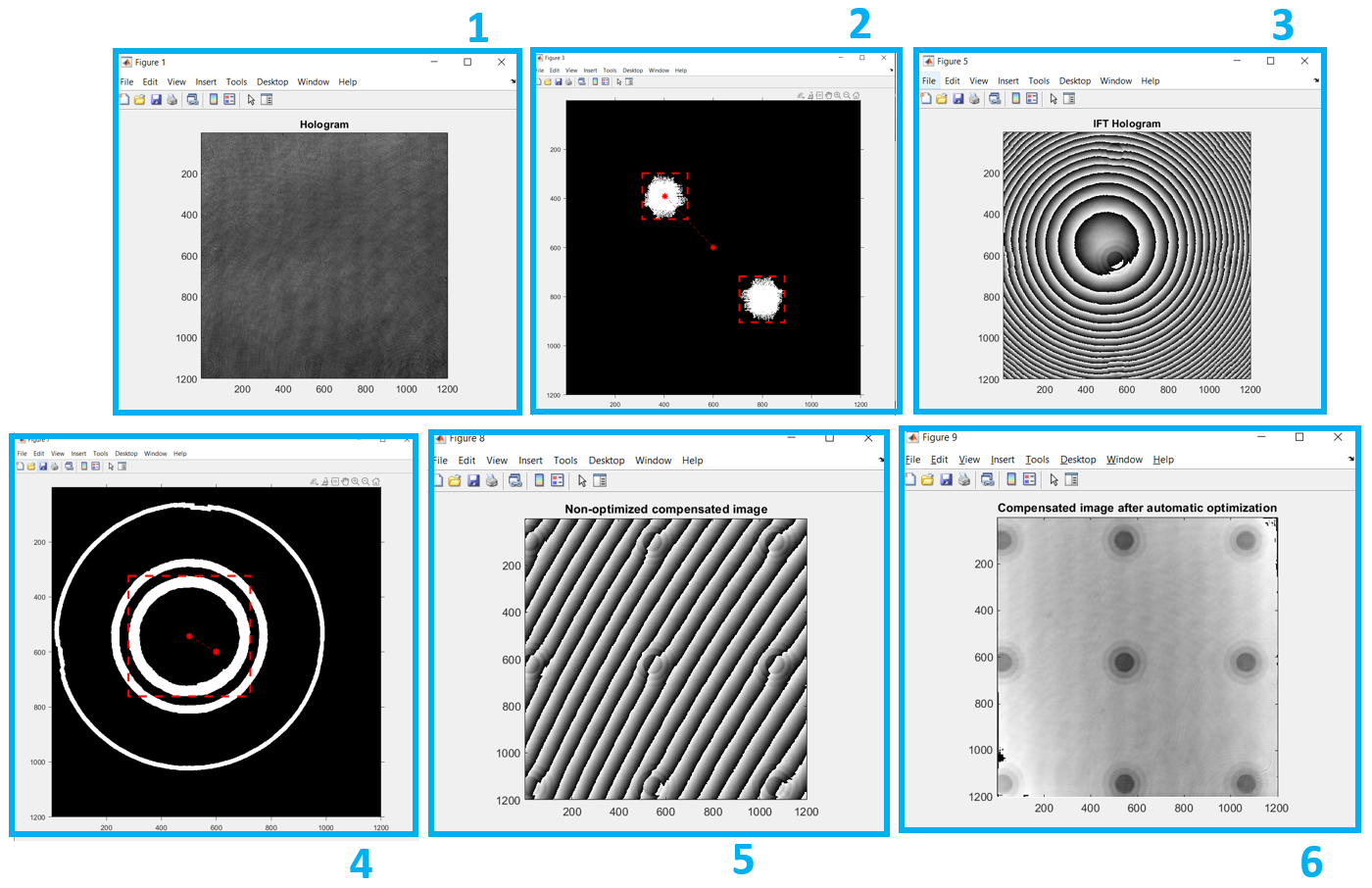


This mode presents the hologram first (1), followed by its binarized Fourier spectrum indicating the automatically selected diffraction orders (2). Next, the phase image compensated for the tilting angle is displayed (3), followed by its binarized image indicating the automatically identified center of the remaining spherical phase factor (4). The non-optimized image (5) is shown after that, and finally, the final compensated image is displayed (6). The accuracy and processing time of the method depend on the complexity of the sample and the selected optimization algorithm.

- - - 1. **Manual mode**

In this mode, the user is first prompted to manually select a region of interest surrounding the +1-diffraction order (1). Then, the user is asked to click on the center of the remaining spherical phase factor (2). The following images illustrate these procedures.


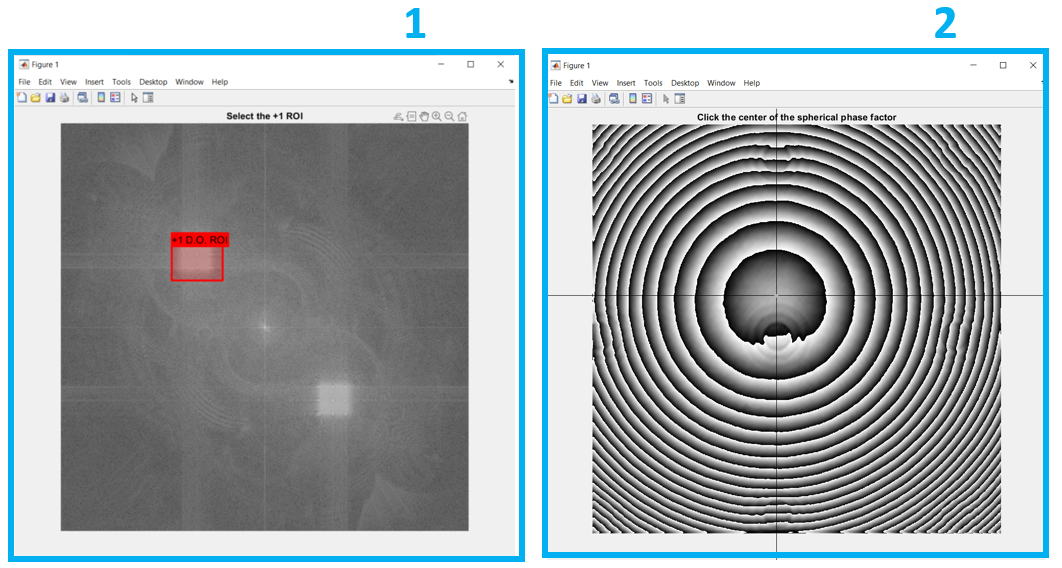


After completing these steps, the user is presented with the final fully compensated image. Although this mode is more robust and generally provides better results, it requires more processing time.


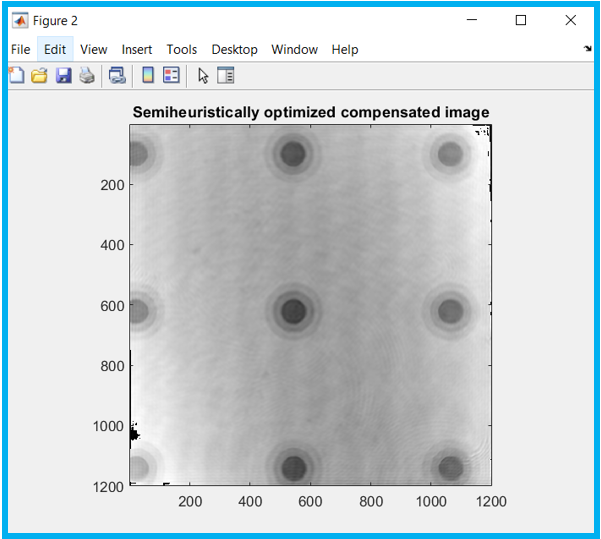


- - 1. **Python scripts**

To execute the phase compensation method, use the 'no_tele_main.py' script, which is the primary operating script, while the 'funs.py' script contains the necessary auxiliary functions. When running the 'no_tele_main.py' script, pay attention to the following lines:

#Loading image file (hologram) to process

user_input = '4cm_20x_bigcakes_square.tif'

filename = 'data/' + user_input

#Variables and flags for hologram reconstruction (Default variables, change accordingly)

Lambda = 633*10**(-9)

dx = 6.9*10**(-6)

dy = 6.9*10**(-6)

#Different available optimization methods (only meeded for automatic method)

#0: FMC 1: FMU 2: FSO 3: SA 4: PTS 5: GA 6: PS 7: GA+PS (See documentation for further details)

algo = 7; #Select method as desired

#Two available cost functions (only needed for automatic method)

#cost = 1 # 0 - BIN -- 1 - SD (See documentation for further details)

cost = 1 #Select function as desired

These variables must be used as they were defined in section 2.1.1. In addition to that, it is necessary to choose the compensation mode that will be used:

###################################################################################

#0: Manual determination of the M&N and H&G coordinates for no-tele compensation.##

#1: Automatic determination of these parameters. ##################################

###################################################################################

auto = 0

Again, this flag variable is defined as for the MATLAB scripts. See section 2.1.1.

- - - 1. **Automatic mode**

In this mode, the user is presented with several intermediate images. As depicted in the following figure.


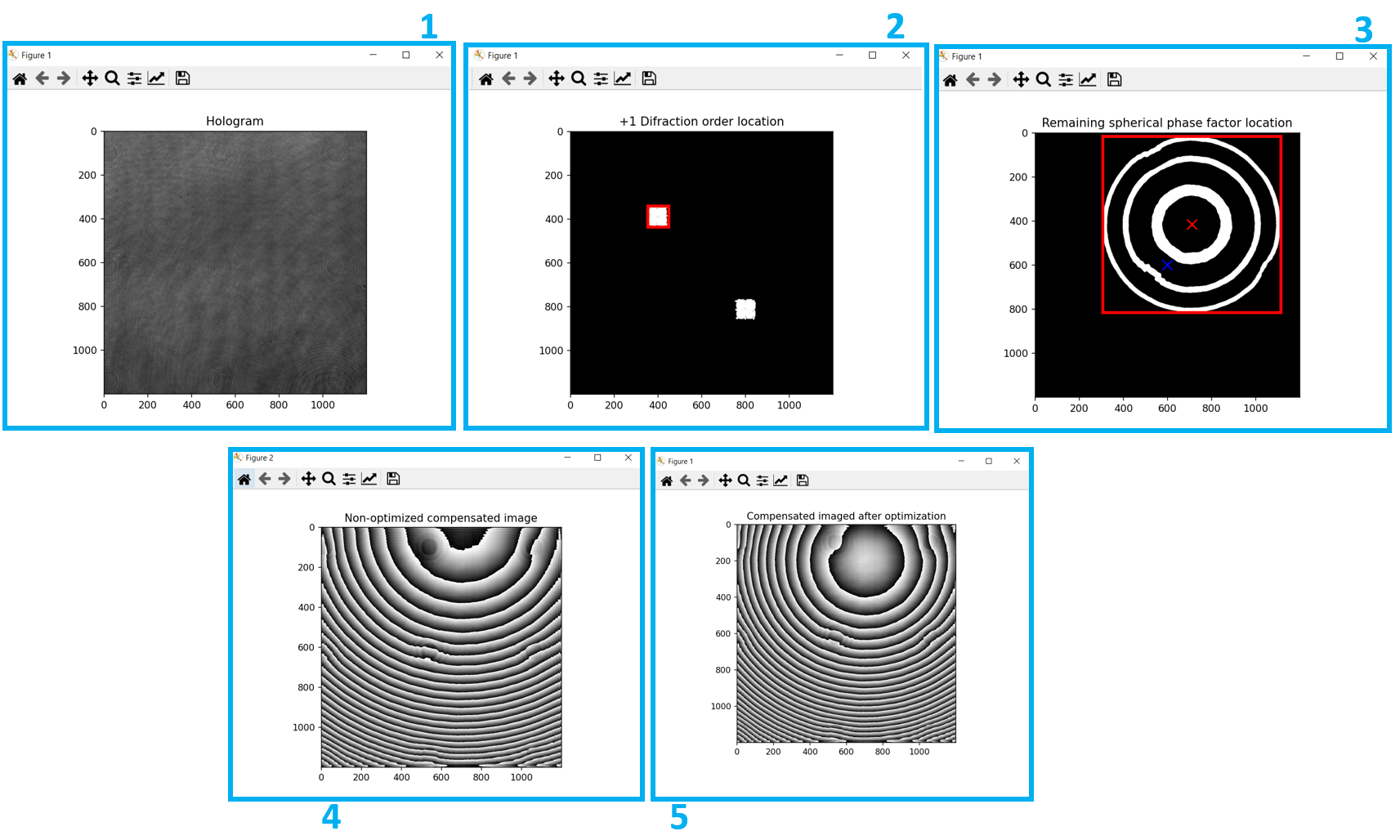


This mode presents the hologram first, followed by its binarized Fourier spectrum indicating the automatically selected diffraction orders. Next, the binarized image indicates the automatically identified center of the remaining spherical phase factor. The non-optimized image is shown after that, and finally, the final compensated image is displayed. The accuracy and processing time of the method depend on the complexity of the sample and the selected optimization algorithm.

- - - 1. **Manual mode**

In this mode, the user is first prompted to manually select a region of interest surrounding the +1-diffraction order (1). Then, the user is asked to click on the center of the remaining spherical phase factor (2). The following images illustrate these procedures.


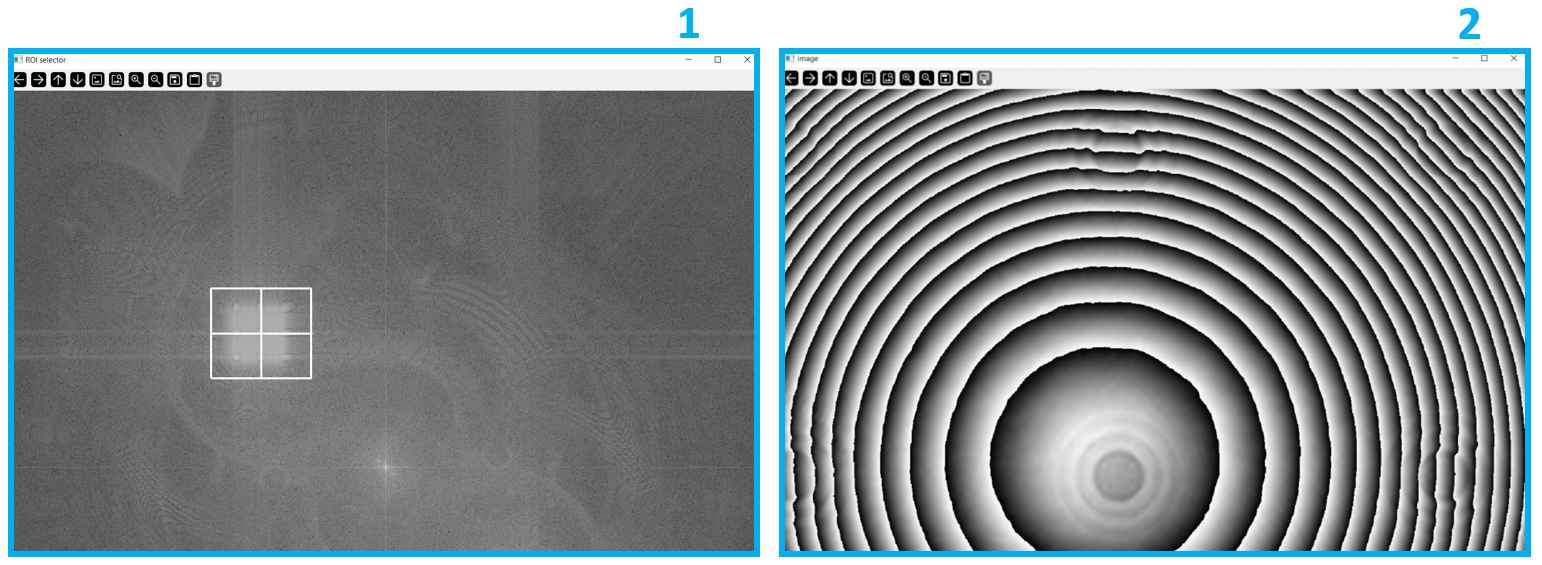


To select the region of interest, the user must press enter after the selection (1). Also, after click over the center of the remaining spherical phase factor, the user must press ‘esc’ (2). After completing these steps, the user is presented with the final fully compensated image. Although this mode is more robust and generally provides better results, it requires more processing time. In many cases, the Python version is at least three times slower than its MATLAB counterpart. This is because MATLAB has default-optimized routines that are not available in Python.


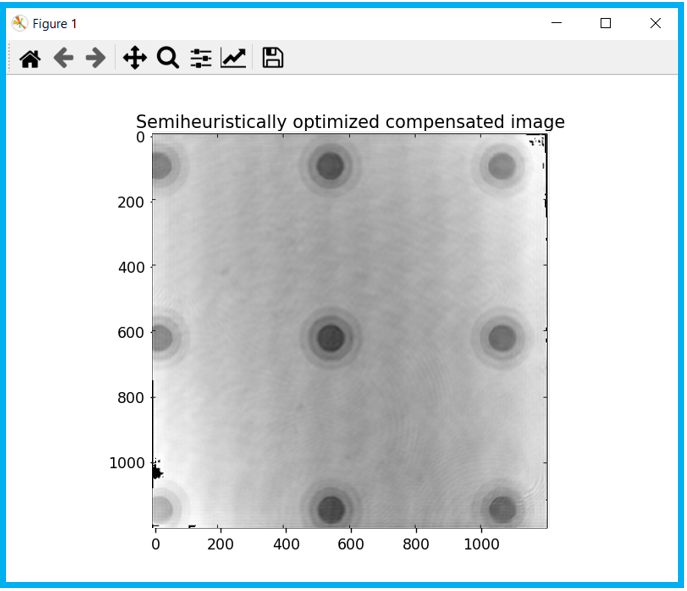


- 1. **GUI version**

This version contains a MATLAB GUI friendly that is suitable for users that need to reconstruct off-axis non-telecentric holograms without knowledge of MATLAB and Python. anyone

- - 1. Installation

To install noteleDHM GUI version, download the .mlapp file, named NonTeleReconApp, from the webpage https://github.com/OIRL/noteleDHM-Tool and open it using MATLAB.

- - 1. Running noteleDHM GUI version

The graphical interface has two tabs: the reconstruction and save tabs. In the reconstruction tab, there are 10 panels , choose hologram (1), visualization of the hologram (2), visualization of the power spectrum (3), parameters (4), manual filtering of the +1 term (5), visualization of the phase image after compensating the interference angle (6), parameters of the spherical wavefront (7), visualization of the fully-compensated phase image (8), fine tuning of the spherical wavefront using minimization (9), unwrapping (10).

**
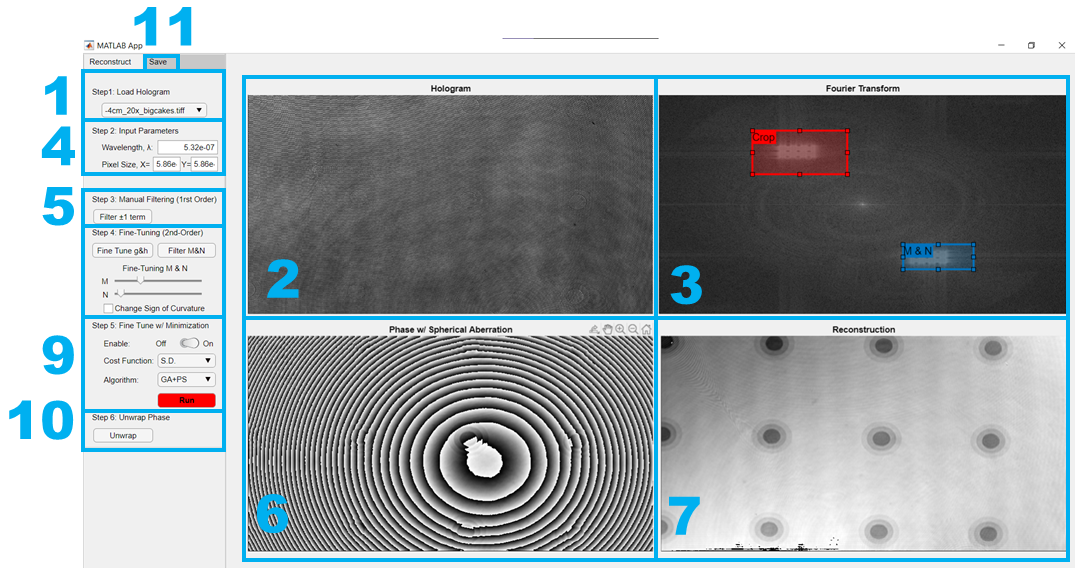
**

1. Load hologram: This drop-down menu allows to select the hologram image file one wants to reconstruct. This file must be located in the same folder as the NonTeleReconApp.mlapp file.

2-3. Visualizations: These two panels display the raw hologram and its Fourier transform, respectively, as soon as the hologram has been loaded.

4. Parameters: In this panel, the user must insert the values of the parameters used to record the hologram: *Wavelength*, which is the source’s wavelength in meters (m), and *Pixel size*, which is the size of the pixel along the X and Y direction in meters (m). By default, the parameters are λ = 532 nm = 5.32e-7 m, and X = 5.86 μm = 5.86e-6 m and Y = 5.86 μm = 5.86e-6 m.

5. Spatial filtering of the +1 order: This panel allows to manual filter the +1 term from the hologram’s spectrum. The user must click the “Filter ±1 term” buttom and draw a rectangle (see red rectangle in panel 3) over the +1 term. The red rectangle is used to filter the hologram’s spectrum and estimate the interference angle (e.g., compensate the linear phase term introduced by the off-axis interference). The user can modify the dimensions of the red rectangle.

6. Visualization of the phase image after compensation of the interference angle. This panel shows the phase image after compensating for the digital reference wave. The phase image is obtained by estimating the angle of the multiplication of the inverse Fourier transform of the filtered hologram’s spectrum with the digital reference wave. The parameters of the digital reference wave are found by the cropped mask defined by the user in step 5. It is important that the center of the remaining spherical wavefront is placed close to the center of the image, play with the dimensions of the red rectangle in step 5.

7. Visualization of the phase image after compensation of the spherical wavefront. This panel shows the phase image after compensating for the spherical wavefront related to using a non-telecentric imaging system. Firstly, this panel shows the reconstructed phase map by compensating a spherical wavefront whose curvature parameters are estimated from the spatial filtered defined by the user and the center of the spherical wavefront estimated in panel 6.

8. Compensation of the spherical wavefront (manual fine-tuning). This step is optional if the selection of the +1 term in step 5 is good enough. However, it is more convenient to use the tools in step 5 to get a preliminary estimation which is then refined with the widgets in this step. Here, the user can define the center of the spherical wavefront in panel 6 by clicking “Fine tune g&h” buttom, allowing the user to adjust the spherical aberration’s center's position manually. The second button, “Filter M&N”, enables the user to draw a second rectangle on panel 3. The implementation only takes account the size of the second rectangle (blue rectangle in panel 3), so one can use the -1 term to find the best dimension by compensating for the spherical wavefront in panel 7. Alternatively, the user can fine tune the M&N parameters using the sliders if the original crop rectangle created in step 5 (red rectangle in panel 3) is close to the ground truth.

9. Fine tuning w/ Minimization (Optional). This is an alternative step to compensate for the spherical wavefront. The user can perform the fine-tuning of the curvature of the spherical wavefront by minimization of a cost function.. The success of the minimization algorithm depends on how close the initial seed already is. As a general rule, a spherical wavefront with one-two ring of curvature should work fine, see below image. To use the minimization algorithm, the user must flip the “Enable” switch. Once flipped, the user should select the cost function and the minimization algorithm. There are two options for the cost function. Option one (Binarization), the cost function measures the total number of phase wraps in the binary reconstructed phase image. Option 2 (S.D.), the cost function calculates the total standard deviation of the compensated phase map. There are seven minimization algorithms: FMC, FMU, FSO, SA, PTS, GA, and PS. A short description of these algorithms is found in the manuscript. After selecting the cost function and the minimization algorithm, the user must click “Run” buttom (in red in panel 9) to start the minimization algorithm. Depending on the selection of algorithms and how close the initial estimates are to the optimal value, this minimization may take anywhere from 30 second to 2 minutes. The reconstructed phase image is updated in panel 7. After the minimization is performed, the “Enable” switch will allow the user to alternate the reconstruction displayed on panel 7 between the automatic or the manual compensation.


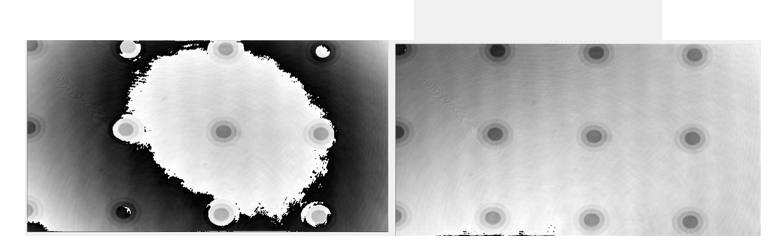


1. Unwrap Phase (Optional). The user can select the estimation and display of the unwrapped phase image for samples in which wrapping is observed. The unwrapping approach is performed through the implementation of the method proposed by Herráez *et. al.* [2,3]. The unwrapped phase image is displayed in panel 7.
2. The Save Tab. The user can save the results shown in the reconstruction panel. In this tab, the user is able to insert the name of the folder (1) to save the .mat files. If the folder is not created, a folder is created with the provided save name in the current directory. In panel 2, the user must insert the name of the file. The user may also specify whether they wish to save the reconstructed phase image provided by the minimization algorithm and the unwrapped phase image. Finally, the “Export Recon” button will create the save file.


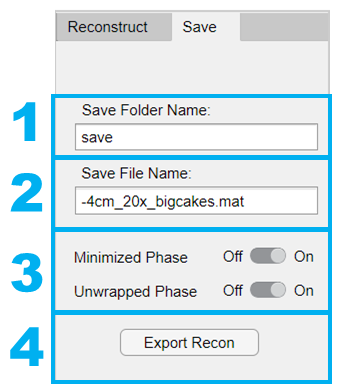


1. **Usage example**

In the GitHub repository, there are 19 holograms. The holograms correspond to five microscopic test targets from the Benchmark Technologies Quantitative Phase Target (QPT^TM^) and a smearing of red blood cells.

The parameters (wavelength, and pixel size) are (0.532 μm, 5.86 μm) for all samples.

Below there is a quick step-by-step procedure for using notele GUI version.

- 1. Open the digital hologram image file.
  2. Introduce the input parameters
  3. Select the +1 term in the hologram’s spectrum; check that the center of the spherical wavefront is centered in panel 6.
  4. Tune the M&N values to remove the spherical wavefront in panel 7. If one fails, please put the reconstructed phase map with a spherical wavefront with reduced curvature (1-2 rings in panel 7).
  5. Enable a minimization algorithm to compensate completely for the spherical wavefront. Click on “Run” bottom.
  6. Save results

A video to show how to use the noteleDHM MATLAB GUI tool is available in YouTube: <https://youtu.be/Hyi5vwgYX7c>.

1. **References**

[1] J. Min, B. Yao, S. Ketelhut, C. Engwer, B. Greve, B. Kemper, Opt. Lett. 42 (2017) 227 - 230. https://doi.org/10.1364/OL.42.000227.

[2] M. A. Herraez, D. R. Burton, M. J. Lalor, and M. A. Gdeisat, "Fast two-dimensional phase- unwrapping algorithm based on sorting by reliability following a noncontinuous path", Applied Optics, Vol. 41, Issue 35, pp. 7437-7444 (2002).

[3] M. F. Kasim, "Fast 2D phase unwrapping implementation in MATLAB", available in https://github.com/mfkasim91/unwrap_phase/.
