# Supplementary material for: Comprehensive tool for a phase compensation reconstruction method in digital holographic microscopy operating in non-telecentric regime
Source: PLoS One. 2023 Sep 8;18(9):e0291103. doi: 10.1371/journal.pone.0291103 (PMC10491004; doi:10.1371/journal.pone.0291103)
Supplement: S1 Appendix — (DOCX) [file pone.0291103.s002.docx]

Appendix A – Minimizing algorithms

The minimization algorithms implemented in the proposed computational software are included in the Optimization and Global Optimization MATLAB toolboxes and the scipy, pyswarms, and parmoo libraries from Python. Below there is a short description of each minimization algorithm tested.

Derivative-based non-linear solvers:

1. FMC method finds the minimum of a constrained multivariable function. *fmincon* in MATLAB and *NonlinearConstraint* in Python’s scipy.

2. FMU approach finds the minimum scalar value of a non-linear unconstrained multivariable objective function. *fminunc* in MATLAB, and *fmin_ncg* in Python’s scipy.

3. FSO solver returns a vector that minimizes the objective function by solving for the function F(x) = 0. *fsolve* in MATLAB and *fsolve* in Python’s scipy.

Non-derivative based solvers:

4. SA is a simulated annealing probabilistic technique well suited for finding the global minimum of a large and discrete search space. *Simmulan-nealbnd* in MATLAB and *dual_annealing* in Python’s scipy.

5. PTS algorithm that finds the points in a Pareto front that minimizes two cost functions of a two-dimensional variable. In this case, we use the J_1_ and J^2^ cost functions. *paretosearch* in MATLAB and *basinhopping* from Python’s scipy.

6. GA algorithm minimizes a cost function given the number of variables in the function by iteratively picking the best population values within the range specified by the bounds. *ga* in MATLAB and *differential_evolution* in Python’s scipy.

7. PS algorithm that does not utilize gradients, allowing for the convergence of cost functions that are not continuous or differentiable. *patternsearch* in MATLAB and minimize with *Nelder-Mead* method in Python’s scipy.
