# Supplementary material for: Comprehensive tool for a phase compensation reconstruction method in digital holographic microscopy operating in non-telecentric regime
Source: PLoS One. 2023 Sep 8;18(9):e0291103. doi: 10.1371/journal.pone.0291103 (PMC10491004; doi:10.1371/journal.pone.0291103)
Supplement: S2 Appendix — (DOCX) [file pone.0291103.s003.docx]

Appendix B – Comparison between the different minimization algorithms

Table B1. Comparison between the performance of each minimization function based on the average processing time, the Percentage Error (PE) of the curvature values (C_x_, and C_y_) and the Structured Similarity Index Measure (SSIM) between the reconstructed phase image for each algorithm and the ground truth phase map obtained after an intensive looping search.

| Metric | FMC | FMM | FMU | FSO | GA | PS | PTS | SA | SGO | GA+PS |
| --- | --- | --- | --- | --- | --- | --- | --- | --- | --- | --- |
| PE C_x_  (mean ± std) | 18.76  ±21.33 | 29.31  ±20.54 | 29.79  ±12.91 | 323.94  ±393.91 | 6.64  ±6.52 | 6.90  ±8.34 | 7.31  ±8.82 | 7.00  ±10.43 | 4.65  ±8.84 | 5.58  ±7.08 |
| Range PE C_x_ | 0.58-58.83 | 9.86-65.78 | 10.73-43.14 | 10.78-1034.40 | 0.27-17.93 | 0.00-18.16 | 0.00-19.20 | 0.14-28.11 | 0.13-19.22 | 0.00-  18.16 |
| PE C_y_  (mean ± std) | 45.41  ±56.30 | 37.53  ±33.51 | 55.69  ±40.64 | 145.11  ±117.82 | 9.93  ±2.27 | 6.19  ±18.20 | 8.64  ±11.59 | 7.42  ±12.51 | 3.09  ±11.81 | 2.38  ±4.86 |
| Range PE C_y_ | 2.80-167.04 | 4.62-97.51 | 9.13-120.00 | 8.46-307.89 | 0.10-6.45 | 0.00-49.71 | 0.00-31.85 | 0.11-31.07 | 0.13-31.86 | 0.00-  12.87 |
| SSIM (mean  ±std) | 0.061  ±0.06 | 0.037  ±0.04 | 0.016  ±0.02 | 0.014  ±0.02 | 0.461  ±0.35 | 0.555  ±0.42 | 0.559  ±0.42 | 0.480  ±0.41 | 0.530  ±0.41 | 0.634  ±0.37 |
| Average Time (sec) | 2.74 | 3.68 | 2.96 | 0.49 | 41.66 | 3.90 | 166.93 | 35.75 | 10.92 | 64.03 |
