# Supplementary material for: Comprehensive tool for a phase compensation reconstruction method in digital holographic microscopy operating in non-telecentric regime
Source: PLoS One. 2023 Sep 8;18(9):e0291103. doi: 10.1371/journal.pone.0291103 (PMC10491004; doi:10.1371/journal.pone.0291103)
Supplement: Attachment — Submitted filename: Response to Reviewers_Final.pdf [file pone.0291103.s004.pdf]

Detailed Response to Reviewers for PONE-D-23-14581: "Comprehensive Tool for a Phase Compensation Reconstruction Method in Digital Holographic Microscopy Operating in Non-Telecentric Regime"

Journal Requirements:

Comment #1 "Please ensure that your manuscript meets PLOS ONE's style requirements, including those for file naming. The PLOS ONE style templates can be found at https://journals.plos.org/plosone/s/file?id=wjVg/PLOSOne_formatting_sample_main_body.pdf and https://journals.plos.org/plosone/s/file?id=ba62/PLOSOne_formatting_sample_title_authors_affiliations.pdf"

Response: We have made formatting changes to ensure that the manuscript meets the PLOS ONE's style guidelines as outlined in the templates previously linked.

Comment #2. "Please note that funding information should not appear in any section or other areas of your manuscript. We will only publish funding information present in the Funding Statement section of the online submission form. Please remove any funding-related text from the manuscript. "

Response: We have removed any mentions of funding information from the revised manuscript, including the paragraph with funding.

Comment #3 "We note that you have stated that you will provide repository information for your data at acceptance. Should your manuscript be accepted for publication, we will hold it until you provide the relevant accession numbers or DOIs necessary to access your data. If you wish to make changes to your Data Availability statement, please describe these changes in your cover letter, and we will update your Data Availability statement to reflect the information you provide."

Response: The repository information has been included in the revised manuscript. We have also updated the Data Availability statement to reflect this.

Comment #4. "PLOS authors have the option to publish the peer review history of their article (what does this mean?). If published, this will include your full peer review and any attached files."

Response: We would like the peer review history to be published once our revised manuscript is accepted.

Response to Reviewers:

The authors thank the anonymous reviewer for reading the manuscript and for his/her effort in providing constructive criticism and positive feedback. A detailed reply to the reviewer's comments is provided below. Our responses are written in blue font below each reviewer's comment, and the corresponding manuscript changes are provided in red font for clarity. We have numbered the reviewer's comments. In cases where numbers were not provided, we have numbered the key points asked so that each point could be addressed clearly.

Reviewer 1

Comment #0: "In the submitted manuscript, the authors present a computational method to process the phase images acquired with a non-telecentric, digital-holographic microscope. The proposed computational method is thoroughly described. Here are some suggestions."

Response: We appreciate the reviewer taking the time to carefully review our manuscript. We have addressed his/her concerns in the revised manuscript.

Comment #1: "In digital holographic microscopy, the phase modulation not induced by the sample can be easily and accurately subtracted using a background image, which is acquired for an empty field of view. The same background image can be used repeatedly for multiple sample images acquired afterward; thus, the process does not increase the data acquisition time much. The computational method proposed by the authors would be useful if it provides the same resolution and phase accuracy as the background subtraction method. That said, I'd strongly recommend the authors to perform a more thorough characterization of the proposed method."

Response: We appreciate the reviewer for this comment. We agree that recording a reference hologram (e.g., background image) without the sample distribution does not increase the acquisition time significantly. Especially, when this reference image can be taken at the beginning of each experiment. To address the reviewer's comment, we have added a new figure (Fig. 8 in the revised manuscript) that compares the background phase values and the resolution between the subtraction method and the proposed one. Figure 8 shows the normalized reconstructed phase images of a USAF phase target for both methods, demonstrating that the accuracy and resolution of both methods is totally comparable. The minimum resolvable element of the USAF phase target is the 9-3 element, which corresponds to 0.78 µm. This value confirms that both methods provide reconstructed images operating at the system's coherent diffraction limit, $d = \lambda/NA = 0.532 / 0.75 = 0.71$ µm. Although the dual-shot subtraction method is simple to perform and computationally inexpensive, its effectiveness is highly dependent on the experimental conditions during data acquisition, requiring that the blank hologram must represent an exact replica of the spherical distortions to produce accurate phase distributions. If the experimental DHM system suffers from external factors such as vibrations and temperature fluctuations, the blank and sample holograms do not have the exact same distortion and the subtraction method will not produce accurate results, requiring the use of computational methods to reduce any residual distortion. This negative result is further exacerbated during the acquisition of large datasets, where the temporal and spatial changes are most likely to occur. However, the proposed method alleviates such constraints by taking advantage of the single shot nature of off-axis DHM.

The following text has been added to the revised manuscript:

L355-377: “Finally, we have compared the performance of the proposed method with the one provided by the subtraction method, which uses a blank hologram to compensate for the spherical aberrations caused by a non-telecentric alignment [21] via the direct subtraction between both reconstructed phase maps. Figure 8 shows the normalized reconstructed phase images of a USAF phase target for the proposed method (Fig. 8a) and the subtraction one (Fig. 8b), demonstrating the high agreement between both methods. The accuracy and resolution of both methods have been evaluated by plotting the normalized phase values along the vertical direction [indicated by the white arrows in Figs. 8(a) and (b)] of the 9 group, see Fig. 8(c). From these profiles, one can identify the minimum resolvable element of the USAF phase target is the 9-3 element, which corresponds to 0.78 μm. This value confirms that both methods provide reconstructed images operating at the system’s coherent diffraction limit, $d = \lambda/NA = 0.532 / 0.75 = 0.71$ μm. Despite that the dual-shot subtraction method is simple to perform and computationally inexpensive, its effectiveness is highly dependent on the experimental conditions during data acquisition, requiring that the blank hologram must represent an exact replica of the spherical distortions to produce accurate phase distributions. If the experimental DHM system suffers from external factors such as vibrations and temperature fluctuations, the blank and sample holograms do not have the exact same distortion and the subtraction method will not produce accurate results, requiring the use of additional computational methods to reduce any residual distortion. This negative result is further exacerbated during the acquisition of large datasets, where the temporal and spatial changes are most likely to occur. However, the proposed method alleviates such constraints by taking advantage of the single shot nature of off-axis DHM.”

The following figures has been added to the revised manuscript:

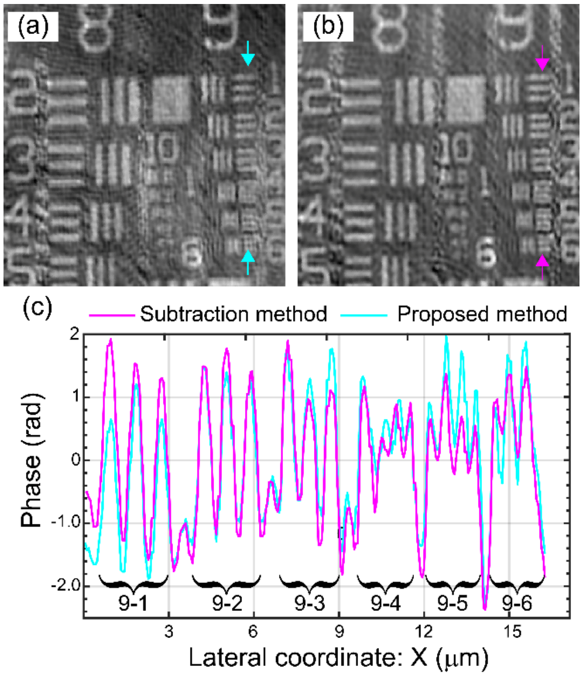

Fig 8. Reconstructed phase images of a USAF phase target using the (a) proposed method and (b) subtraction method. (c) Phase profile along the vertical direction (marked by the color arrows in panels a and b) through the horizontal lines of group 9.

Comment #2: “The resolution measurement has not been performed. This could be done by analyzing the edge of the star-target image (Figure 6).”
Response: We have used the reconstructed phase images of a USAF test target from the Benchmark Technologies Quantitative Phase Target (QPT™) by Benchmark Technologies to measure the resolution of the proposed method. Please see response to comment #1 for more details.

Comment #3: “More important, the phase measurement accuracy needs to be shown using samples of known refractive index values (e.g., polymer microspheres in index-matching liquid).”
Response: We apologize for the miscommunication. The phase measurement accuracy is shown in Fig. 6. This figure shows the reconstructed 2D phase map of a star target from the Benchmark Technologies Quantitative Phase Target (QPT™). The refractive index of the star target is well known and given by the manufacturer (refractive index = 1.52). In particular, panel (b) in Fig 6 shows the radial phase profile at two different radii: r = 43.95 µm (pink), and r = 73.25 µm (cyan). The gray-shaded area shown in Fig. 6(b) marks the nominal phase value based on the manufacturer’s specifications (n = 1.52 and thickness = 350 nm). There is a high similarity between the experimental phase values and the nominal ones, demonstrating the accuracy of the proposed method to provide accurate quantitative phase imaging.
The following text has been rewritten in the revised manuscript:

L314-321: “The nominal phase values have been calculated based on the manufacturer’s specifications, a refractive index and thickness equal to 1.52 and 350 nm, respectively. There is a high similarity between the experimental phase values and the nominal ones, demonstrating the accuracy of the proposed method to compensate spherical phase distortions. The results illustrated in Fig. 6 confirm that, within experimental errors, the spherical phase factor introduced by the non-telecentric configuration has been compensated, providing a linear shift-invariant quantitative phase imaging tool.”

Comment 4: “The low-frequency phase distribution in the background region (i.e., non-sample region) would be useful to confirm the effectiveness of the proposed method.”
Response: We agree with the reviewer, and, for this reason, quantitative phase profiles along different background regions have been included in Fig. 7, see non-sample profiles colored in green, cyan, magenta. The comparison of these background profiles in Figs. 7b&d confirms that the low frequency phase information is fairly uniform across the different directions, demonstrating the effectiveness of our proposed method to compensate spherical distortions in all directions.

The following sentence has been added in the revised manuscript:
L330-335: “We have plotted some vertical and horizontal background phase profiles in Figs. 7 (b) and (d) measured at the colored lines over the phase images in Fig 7(a) and (c). The comparison of these background profiles in Figs. 7(b) and (d) confirms that the low frequency phase information is fairly uniform across the different directions Aside from minimal discrepancies from a complete flat background, these results demonstrate the effectiveness of our proposed method to compensate spherical distortions in all directions.”

Reviewer 2

Comment #0: “The authors report about a method for compensation of phase aberrations in quantitative phase imaging (QPI) with non-telecentric off-axis digital holographic microscopy (DHM). After an explanation of the underlying principles and characterization of the method by utilizing a phase test chart the application on a technical sample and red blood cells is illustrated. In general, the manuscript is motivated, organized, and includes adequate references. The experimental investigations appear to be accurately performed. The results are plausible. The authors address an important topic in QPI with DHM: The compensation of spherical phase aberrations which may be of interest for the field of DHM and the interdisciplinary areas of QPI and label-free biomedical imaging. In summary, the content of the manuscript appears to be suitable for the journal PLOS one. However, the authors should consider revisions.”

Response: We appreciate the kind words from the reviewer. We hope to have adequately addressed the reviewer's comments in the revised manuscript.

Comment #1: “Abstract: From the abstract the novelty aspects of the proposed phase compensation concept with respect to reference 35 becomes not fully clear. The authors should clarify the abstract concerning this topic.”

Response: We believe that the novelties of our contribution with respect to Ref. [35] are the following. Firstly, the proposed method further simplifies the algorithm initially reported by Ref. [35] by reducing the number of required parameters to only the camera pixel size and light source wavelength. Secondly, we provide a step-by-step guide for the practical implementation of the proposed method with specific design choices made explicit (i.e., toolboxes, functions, code, etc.) for each step. We believe that the proper implementation of a framework is critical to achieve successful results. Thirdly, the proposed algorithm offers an additional advantage by incorporating an optional minimization algorithm for quantitative reconstructed phase images. This thoughtful inclusion aims to alleviate the user's burden, streamlining the process and making it more user-friendly. Finally, but not least important, we have provided an open-access computational tool accessible to the public with code and application (GUI). The developed software tool will allow researchers in life and material sciences, even those without computational reconstruction knowledge, to analyze their results accurately, leading to new discoveries.

We have rewritten the abstract in the revised manuscript to clarify the novelties of our contribution:

“Quantitative phase imaging (QPI) via Digital Holographic microscopy (DHM) has been widely applied in material and biological applications. The performance of DHM technologies relies heavily on computational reconstruction methods to provide accurate phase measurements. Among the optical configuration of the imaging system in DHM, imaging systems operating in a non-telecentric regime are the most common ones. Nonetheless, the spherical wavefront introduced by the non-telecentric DHM system must be compensated to provide undistorted phase measurements. The proposed reconstruction approach is based on previous work from Kemper’s group. Here, we have reformulated the problem, reducing the number of required parameters needed for reconstructing phase images to the sensor pixel size and source wavelength. The developed computational algorithm can be divided into six main steps. In the first step, the selection of the +1-diffraction order in the hologram spectrum. The interference angle is obtained from the selected +1 order. Secondly, the curvature of the spherical wavefront distorting the sample's phase map is estimated by analyzing the size of the selected +1 order in

the hologram's spectrum. The third and fourth steps are the spatial filtering of the +1 order and the compensation of the interference angle. The next step involves the estimation of the center of the spherical wavefront. An optional final optimization step has been included to fine-tune the estimated parameters and provide fully compensated phase images. Because the proper implementation of a framework is critical to achieve successful results, we have explicitly described the steps, including functions and toolboxes, required for reconstructing phase images without distortions. As a result, we have provided open-access codes and a user interface tool with minimum user input to reconstruct holograms recorded in a non-telecentric DHM system."

Comment #2: "Introduction: To complete the description of the-state-of-the-art the authors may consider adding that non-telecentric arrangements can simplify the combination of DHM with commercial optical microscopes as, for example, reported in Drug Deliv. and Transl. Res. 12, 2207–2224 (2022)."

Response: We appreciate the suggestion and have included the mention citation as part of the introduction when mentioning potential reasons for non-telecentric arrangements.

The following sentence has been added to the revised manuscript:

L72-74: "The third reason is related to the integration of DHM imaging modality with commercial microscopic systems to extend the reach of the DHM technique to a broader community and enable dual-mode fluorescent imaging with QPI [20]."

The following reference has been added to the revised manuscript

"20. A. Marzi, K. Eder, A. Barroso, A. Wågbrø, Y. Mørch, A. Hatletveit, T. Visnes, et al., Drug Deliv Transl Res 12(9) (2022), 2207-2224. https://doi.org/10.1007/s13346-022-01207-5."

Comment #3: "Introduction: In the last paragraph of the introduction the novelty aspects/extensions of the proposed phase compensation concept with respect to reference 35 become not fully clear. The authors should consider adding further clarifying details."

Response: Following the reviewer's comment and the novelties of our work described in Comment #1, we have rewritten the introduction in the revised manuscript.

L118-139: "In 2017, Min et al. proposed a single-shot computational approach to estimate the spherical wavefront of non-telecentric DHM systems based on a spectral analysis of the recorded holograms [36]. This work extends Min's approach by further reducing the number of required parameters needed for reconstructing phase images. The input parameters of our computational method are the sensor's pixel size and the wavelength of the light source. Because the proper implementation of a framework is critical to achieve successful results, the accuracy of the algorithm proposed by Min et al. is highly dependent on the methods used for the thresholding and segmentation of the ±1 diffraction terms and their compact support size. A preliminary computational function to reconstruct non-telecentric holograms was recently implemented in the pyDHM library under the CNT function [37]. This function provides reconstructed phase images without or with minimum phase distortions after finding the best curvature of the spherical wavefront along the two lateral spatial coordinates using two nested for loops, leading to a high processing time. However, in this work, we have explicitly described the steps, including functions and toolboxes, required for reconstructing phase images without distortions. In contrast to Min's method, we have included iterative minimization algorithms [18] as an optional final optimization step to fine-tune the estimated parameters of the spherical wavefront and provide fully compensated phase images. The implementation for each step of

the proposed method has been thoughtfully investigated, aiming to develop a generalized computational tool in DHM. Our approach is independent of the sample's size (i.e., not requiring sample-free field of view within the hologram). Additionally, we validate the performance of this approach for several microscopic samples, including biological and non-biological samples, distorted with a spherical wavefront with different curvature. The proposed method has been implemented in MATLAB 2021a and Python 3.7.1 and is publicly available via GitHub [38], offering an open-source reconstruction tool (i.e., codes and GUI) for the DHM community."

Comment #4: "Section 2: The authors may consider removing "operating" from the section title."
Response: The title from Section 2 has been changed to "Off-axis Digital Holographic Microscopy in non-telecentric mode."

Comment #5: "Section 2: In general, the explanations in this section include many details and are partly difficult to understand. The author may consider shortening and clarifying the text and to further emphasize the most important statements/topics."
Response: We appreciate the reviewers' suggestions regarding Section 2. However, we believe that this section should be preserved as it is. Whereas this section may be quite dense, the details explained on it are important for new researchers (i.e., graduate students). This is especially important since there are not reported works providing this information for non-telecentric systems. One of the goals for our research groups is increasing the knowledge in Optical Engineering, enhancing career opportunities for undergraduate and graduate students, including underrepresented students. Nonetheless, if the reviewer still believes that this section is to complex, we are happy to reduce it and provide this information as an appendix.

Comment #6: "Section 2: Fig. 1: The authors show the sketch of a specific experimental arrangement in which sample illumination and reference wave are plane waves. However, in practice, e.g., a sample illumination via a condenser lens (for illustration see, e.g., ref. 35 of the manuscript or Drug Deliv. and Transl. Res. 12, 2207–2224 (2022)) or utilization of a spherical reference wave can also result in a spatial frequency spectrum as illustrated in Fig. 2 of the manuscript. The authors thus may consider adding a discussion concerning the possible transfer of their approach to a more general regime."
Response: We appreciate the reviewer's comment. The performance of the proposed approach is not restricted to non-telecentric imaging systems and reference plane waves. Our computational methods can be used for any off-axis DHM system in which a spherical wavefront distorts the complex object distribution in the recorded hologram. This distorting spherical wavefront can come from the object illumination, the reference illumination, the imaging system or all the above. We have validated this statement by introducing a non-telecentric relay system in the reference arm of our off-axis Mach Zehnder DHM system (Fig. 1 in the manuscript) and reconstructing the phase image with our computational tool. Even though, the object distribution is now affected by an additional spherical phase factor coming from the object, we were able to remove it properly.

The following sentences have been changed in the revised manuscript:
L82-83: "Spherical wavefront distortions must be compensated to provide accurate phase measurements across the imaged field of view, converting the non-telecentric DHM system into a linear shift-invariant phase tool. These distortions can arise from several different sources and can be compensated via physical or numerical methods."

L468-452: "Although the proposed computational approach has been validated with non-telecentric DHM imaging systems and reference plane waves, it can be used for any off-axis DHM system in which a spherical wavefront distorts the complex object distribution in the recorded hologram. This distorting spherical wavefront can come from the object illumination, the reference illumination, the imaging system or all the above."

Comment #7: "Section 3: Figs. 3 and 5 (major point): From the explanations it becomes not fully clear how the spherical phase aberrations are compensated and what are the differences in the procedure reported in reference 35 of the manuscript. For, example: Is equation 10 multiplied or subtracted from phase images like shown in Fig. 5a? The authors should add substantial clarifying information concerning this topic (and perhaps may extend Fig. 4 for an additional illustrating/clarifying sub figure?)."
Response: We apologize for the confusion. Please see reply comment #1 to understand the main difference between Ref. [35] and this work. Figure 5a, which is the reconstructed phase image distorted by a spherical wavefront, is obtained by multiplying the complex amplitude distribution of the inverse Fourier Transform of the filtered hologram spectrum [Eq. (5)] with the complex amplitude distribution of the digital reference wave [Eq. (10)]. We have remade Fig 4 to clarify this confusion.

Figure 4 has been modified in the revised manuscript:

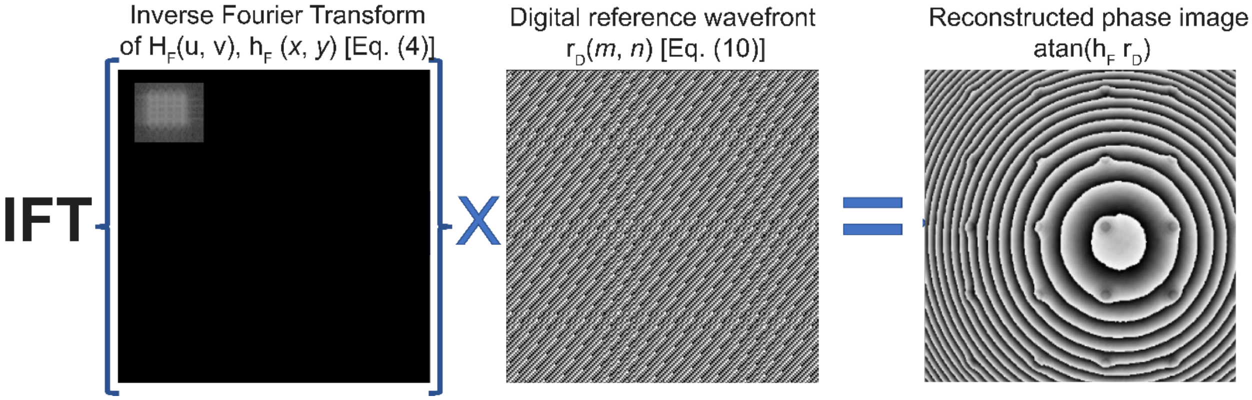

"Fig 4. Compensation of the interference angle by multiplying the inverse Fourier transform (IFT) of the filtered hologram's spectrum [h_F, Eq. (4)] with a digital reference wave (r_D). A spherical wavefront related to using a non-telecentric imaging system still distorts the reconstructed raw phase image."

Comment #8: "Section 3: Figs. 7 and 8: The investigated sample "wedding cakes" should be explained with more details. The authors may consider adding cross-section plots through the phase images in Figs. 8c and 9a."
Response: We agree that a cross-sectional profile is a useful way of visualizing the "wedding cakes" sample. We have included a cross-sectional plot along the pink direction of Fig. 9c in Fig. 9e. This direction has been selected to highlight that the phase values in the wedding cakes are the same, independently of the field of view.

The panel (e) has been added to the following figure:

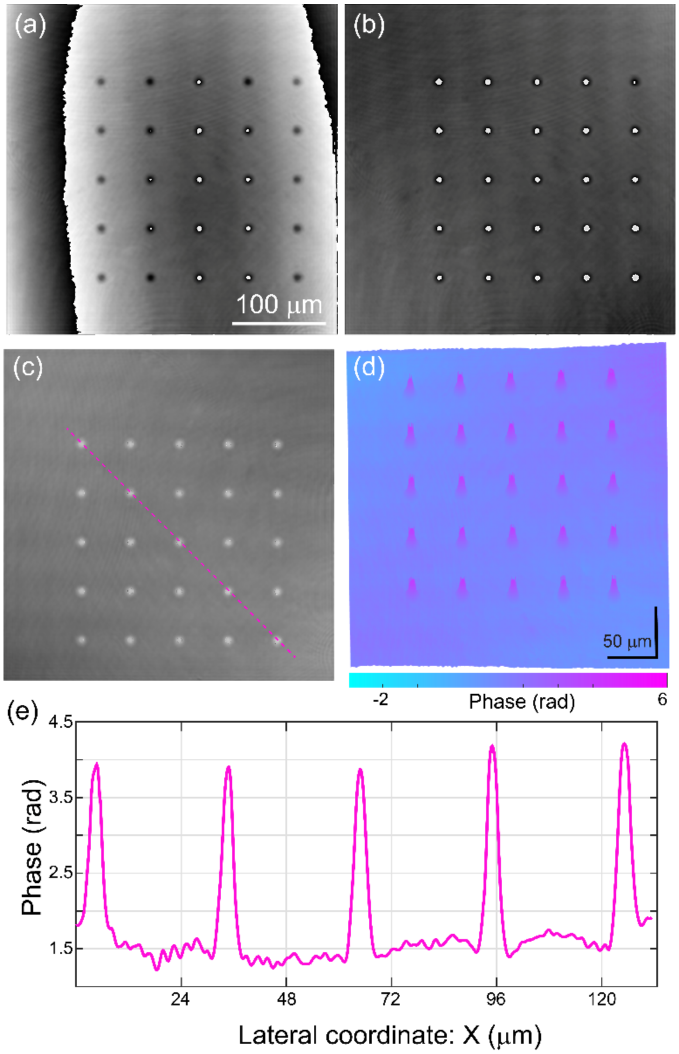

Fig 9. (a-b) Reconstructed phase images of wedding cakes before (a) and after (b) applying a minimization algorithm to computationally remove any distorting linear and spherical phase aberrations. (c) Unwrapped reconstructed phase image. (d) Three-dimensional pseudo color image of the phase map shown in panel (c). (e) Cross-sectional profile view of phase along the pink direction in panel (c).

Comment #9: "Section 3: 2nd paragraph below Fig. 7: The description concerning the minimization algorithms should be supported by data, perhaps, in a figure or table."

Response: We appreciate the feedback and agree a table would be useful in comparing the different minimization approaches. We have included these results as Appendix B and will briefly discuss them in the manuscript.

The following changes has been made in the revised manuscript:

L407-431: "The comparison between the different minimization algorithms is Table B1 in the Appendix B. Two metrics are used to evaluate the performance of these algorithms using the reconstructed phase images. These metrics are the Percent Error (PE), the Structured Similarity Index Measure (SSIM), and the average time. The processing time is reported based on a Windows-based i7-8700 K CPU (3.70 GHz) 16.0 Gbyte RAM desktop computer. The PE compares the estimated curvature of the spherical wavefront along the two lateral directions (C_x,

and C_y) obtained after applying the minimization algorithm and the manual estimation of those parameters using an iterative looping. For example, if the estimated C_x value using the GA minimization algorithm is equal to 0.5769, and the manual estimation of C_x is 0.5707, the PE value is equal to 1.08% (e.g., PE = |(0.5769-0.5707)|/ 0.5707 × 100%). The SSIM metric compares the reconstructed phase image for each algorithm and the ground truth phase map obtained after the intensive iterative looping. For a complete statistical analysis, Table 1B reports the mean and the standard deviation values of the PE and SSIM metrics as well as the range of the PE metric within the tested experimental holograms. The results in Table 1B shows that the combination of the GA and PS minimization algorithms provides the highest similarity between the reconstructed phase images obtained after the minimization process and the ground truth phase image with an average SSIM value of 0.634 and a standard deviation (std) of 0.37. On average, the hybrid GA+PS approach takes approximately a minute per image to find the C_x and C_y values and reconstruct phase images with minimum phase distortions. Although the fastest minimization algorithm is the FMU function with an average processing time of 2.96 seconds, the reconstructed phase images present significant phase distortions, leading to an almost null SSIM value. The PS algorithm is also quite fast, taking about 4 seconds on average to find the minimum values. Nonetheless, the similarity between the reconstructed phase images is slightly reduced from 0.634 with the GA+PS approach to 0.555 with the PS method."

The following table has been added in Appendix B.

"Table B1. Comparison between the performance of each minimization function based on the average processing time, the Percentage Error (PE) of the curvature values (C_x, and C_y) and the Structured Similarity Index Measure (SSIM) between the reconstructed phase image for each algorithm and the ground truth phase map obtained after an intensive looping search. Minimization functions: FMC: constrained multivariable function; FMM: minimax; FMU: non-linear unconstrained multivariable objective function; FSO: fsolver; GA: genetic algorithm; PS: pattern search; PTS: pareto search; SA: simulated annealing; SGO: surrogate optimization search; and GA+PS: hybrid genetic algorithm and pattern search."

Metric	FMC	FMM	FMU	FSO	GA	PS	PTS	SA	SGO	GA+PS
PE C_x (mean ± std)	18.76 ±21.33	29.31 ±20.54	29.79 ±12.91	323.94 ±393.91	6.64 ±6.52	6.90 ±8.34	7.31 ±8.82	7.00 ±10.43	4.65 ±8.84	5.58 ±7.08
Range PE C_x	0.58-58.83	9.86-65.78	10.73-43.14	10.78-1034.40	0.27-17.93	0.00-18.16	0.00-19.20	0.14-28.11	0.13-19.22	0.00-18.16
PE C_y (mean ± std)	45.41 ±56.30	37.53 ±33.51	55.69 ±40.64	145.11 ±117.82	9.93 ±2.27	6.19 ±18.20	8.64 ±11.59	7.42 ±12.51	3.09 ±11.81	2.38 ±4.86
Range PE C_y	2.80-167.04	4.62-97.51	9.13-120.00	8.46-307.89	0.10-6.45	0.00-49.71	0.00-31.85	0.11-31.07	0.13-31.86	0.00-12.87
SSIM (mean ±std)	0.061 ±0.06	0.037 ±0.04	0.016 ±0.02	0.014 ±0.02	0.461 ±0.35	0.555 ±0.42	0.559 ±0.42	0.480 ±0.41	0.530 ±0.41	0.634 ±0.37
Average Time (sec)	2.74	3.68	2.96	0.49	41.66	3.90	166.93	35.75	10.92	64.03
